# The innate thermogenic capacity of brown adipose tissue develops independently of sympathetic signaling

**DOI:** 10.1016/j.molmet.2025.102299

**Published:** 2025-12-09

**Authors:** Ethan C. Fein, Sarmistha Mukherjee, Joseph A. Baur, Patrick Seale

**Affiliations:** 1 Institute for Diabetes, Obesity & Metabolism, Perelman School of Medicine, University of Pennsylvania, Philadelphia, PA, 19104, USA; 2 Department of Cell and Developmental Biology, Perelman School of Medicine, University of Pennsylvania, Philadelphia, PA, 19104, USA; 3 Department of Physiology, Perelman School of Medicine, University of Pennsylvania, Philadelphia, PA, 19104, USA

**Keywords:** Brown adipose tissue, Brown adipocyte, Ucp1, Sympathetic nervous system, Beta adrenergic signaling

## Abstract

Brown adipose tissue (BAT) dissipates energy as heat in response to β-adrenergic signaling induced by the sympathetic nervous system (SNS). While this pathway is essential for the cold-induced remodeling and metabolic activity of BAT, its role in developmental programming is unclear. Here, we show that brown adipocytes acquire thermogenic identity during embryogenesis independently of sympathetic innervation and β-adrenergic signaling. Genetic sympathectomy or disrupted β-adrenergic signaling had minimal effects on thermogenic gene expression or tissue morphology during either embryonic or postnatal BAT development in the absence of cold stress. Functional analyses revealed that the SNS is likely required for circulatory support of BAT activity during β-adrenergic stimulation but not for the development of the thermogenic capacity of BAT itself. These findings demonstrate that developmental and cold-responsive BAT remodeling are mechanistically distinct processes. Defining the molecular programs that drive BAT development may reveal new strategies to enhance BAT formation and function without relying on β-adrenergic stimulation.

## Highlights

• Brown adipose tissue (BAT) acquires its thermogenic identity during fetal development in mice.
• BAT becomes densely innervated by sympathetic fibers during fetal development.
• The thermogenic identity of brown adipocytes develops independently of sympathetic innervation or β-adrenergic signaling.
• The SNS supports BAT activity during β-adrenergic stimulation but is dispensable for establishing its thermogenic capacity.

## 1 Introduction

Obesity arises when caloric intake exceeds energy expenditure. Current pharmacologic strategies for obesity largely function by suppressing food intake. Complementary approaches that enhance energy expenditure have the potential to further improve metabolic outcomes and promote more durable weight loss. One promising target is brown adipose tissue (BAT), which is specialized for heat generation (thermogenesis) through energy expenditure [1,2]. In brown adipocytes, thermogenesis is achieved by the uncoupling of the mitochondrial proton gradient from ATP production, mediated by the BAT-specific uncoupling protein 1 (UCP1) and via the activity of other ATP-dependent pathways [3]. The therapeutic potential of BAT is currently limited by its relative scarcity and dormancy in humans, highlighting the need to better understand how BAT develops [[4], [5], [6], [7]].

In mice, brown adipocytes first appear in the interscapular region around embryonic day 15 (E15) [[8], [9], [10]]. Over the next ∼4 days before birth, brown adipocytes upregulate expression of uncoupling protein 1 (UCP1) and gain the capacity for mitochondrial uncoupling [[11], [12], [13]]. However, it is not known what extrinsic signals, if any, stimulate this development. Canonically, the predominant physiologic stimulus controlling the differentiation and metabolic activity of brown adipocytes is the cold-induced release of norepinephrine (NE) from the sympathetic nervous system (SNS) [3]. SNS-mediated stimulation of BAT by NE is induced when environmental temperature falls below the thermoneutral point (∼30 °C for adult mice) [3]. NE affects brown adipocytes primarily through β-adrenergic receptors, resulting in activation of the associated G protein (G_s_) and cyclic AMP (cAMP) production [3]. cAMP-dependent protein kinase A (PKA) induces the release of free fatty acids (FFAs) from stored triglycerides [14,15]. The liberated FFAs allosterically activate UCP1 and serve as metabolic substrates for thermogenesis [[16], [17], [18]]. cAMP-dependent signaling pathways also promote the transcription of key thermogenic genes such as *Ucp1*, *Dio2*, and *Ppargc1a* [19,20]. In addition to effects on existing adipocytes, sympathetic activity expands the population of brown adipocytes by promoting the proliferation and differentiation of adipogenic progenitor cells [3,[21], [22], [23], [24], [25]].

Ablation of sympathetic signaling in BAT causes dysregulated lipid metabolism, decreased cold-stimulated blood flow and glucose uptake, and decreased proliferation of progenitor cells [23,[26], [27], [28], [29], [30], [31], [32], [33], [34]]. The BAT-intrinsic defects are accompanied by increased susceptibility to diet-induced obesity and cold-induced hypothermia [[33], [34], [35]]. Expression of UCP1 remains detectable in denervated BAT, suggesting that BAT possesses intrinsic thermogenic character [31,34,36]. Because most studies have been conducted at room temperature or below, these phenotypes reflect the importance of the SNS for cold-responsive BAT remodeling and function. However, the contribution of the SNS to BAT development is currently unknown.

The SNS is essential for several aspects of development in mice, including for fetal survival [37,38] and for the biogenesis of the heart [39], pancreatic islets [40], and hematopoietic stem cells [41]. More broadly, peripheral nerves have important roles in the development of the lungs [42], salivary glands [43], and bone [44].

In this work, we investigated the contribution of the SNS to the developmental programming of BAT. Using genetic models that either ablate sympathetic nerves or block β-adrenergic signaling in adipocytes, we found that thermogenic gene expression and cellular morphology in embryonic and postnatal BAT develop normally in the absence of sympathetic innervation or β-adrenergic signaling, provided mice are reared under thermoneutral conditions. Functional studies revealed that the intrinsic thermogenic capacity of BAT is preserved despite genetic ablation of sympathetic innervation. These findings demonstrate that sympathetic signaling is dispensable for the developmental establishment of BAT identity and function, emphasizing the importance of cell-intrinsic mechanisms in BAT development.

## 2 Results

### 2.1 The thermogenic program is activated prenatally in mice

**Figure 1:**
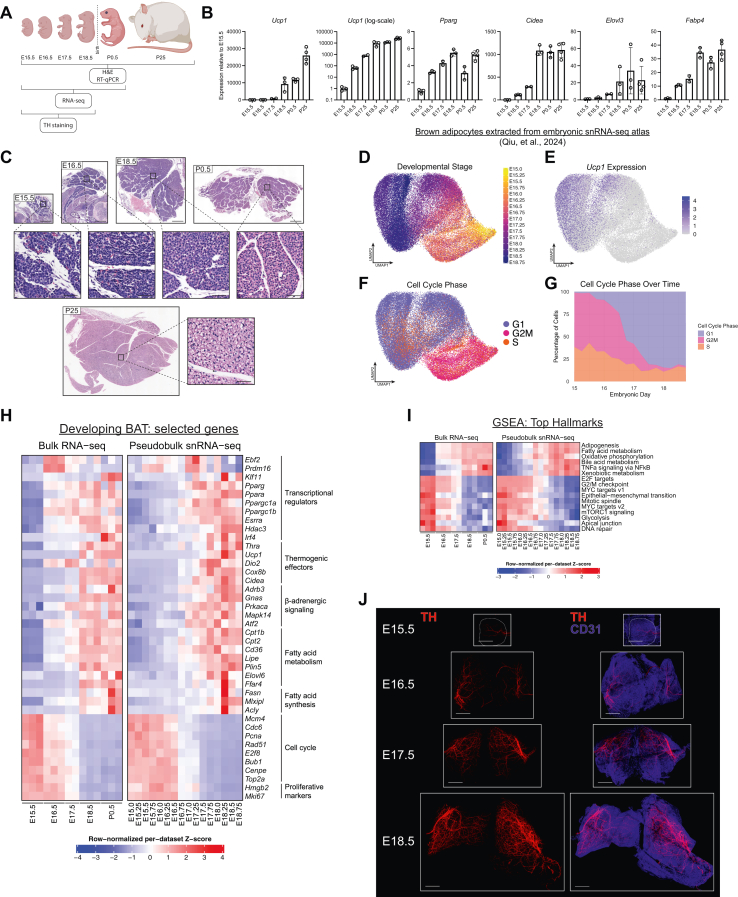
**The thermogenic program is activated prenatally in mice** (**A)** Analysis of BAT from wild-type mice at embryonic day 15.5 (E15.5) through E18.5, P0.5, and P25. **(B–C)** mRNA expression levels (B) and H&E staining (C) of BAT during late embryogenesis and early postnatal life (*n* = 2–3 per timepoint; mean ± SD). Scale bars: 500 μm (tissue), 50 μm (inset). **(D)** UMAP of brown adipocyte nuclei computationally extracted from an embryonic snRNA-seq atlas of murine embryonic development [45], colored by developmental stage. **(E**–**G)** Expression of *Ucp1* (E) and computationally predicted cell cycle phase (F, G) of embryonic brown adipocyte nuclei. **(H–I)** Heatmap of normalized expression levels of selected genes (H) or ssGSEA scores (I) in bulk RNA-seq of BAT (this study) or snRNA-seq of brown adipocytes at the indicated timepoints. Gene sets were ranked by normalized enrichment score (NES) based on E18.0-E18.75 vs. E15.0-E15.75 pseudobulk snRNA-seq data. All pathways with an adjusted *p* value < 0.01 are shown. **(J)** Whole-mount immunostaining of TH (red) and CD31 (blue) in cleared embryonic BAT at the indicated embryonic timepoints. Scale bars: 500 μm.

To characterize fetal and early postnatal BAT development, we dissected BAT from embryos spanning embryonic days 15.5–18.5, pups on the first day of life (postnatal day 0.5; P0.5), and adolescent mice at 25 days old (P25) (Figure 1A). Expression of classic BAT-related mRNAs, including *Ucp1*, *Pparg*, *Cidea*, *Elovl3*, and *Fabp4*, markedly increased from E15.5 to E18.5, suggesting that late embryogenesis is a period during which brown adipocytes rapidly acquire their molecular identity (Figure 1B). In particular, expression of *Ucp1* increased exponentially (∼10,000-fold) over this period, reaching levels comparable in magnitude to those in adolescent mice (Figure 1B). H&E staining revealed progressive morphologic differentiation of BAT, including maturation of the lobular architecture and the appearance of small lipid droplets at P0.5 (Figure 1C).

To broadly analyze gene expression changes in developing brown adipocytes, we utilized a publicly accessible single-nucleus RNA-seq (snRNA-seq) atlas of mouse development, consisting of several million sequenced nuclei from embryos staged at 6-hour (0.25-day) intervals from E8 to P0 [45]. We extracted 113,999 nuclei annotated as adipocytes or adipocyte precursor cells from E15.0-P0. We then further subsetted the data to obtain 52,100 nuclei from E15.0-E18.75 with gene expression profiles characteristic of developing brown adipocytes, rather than preadipocytes or white adipocytes (Figure 1D). Consistently with the RT-qPCR results, *Ucp1* expression increased along developmental time from E15.0-E18.75 (Figure 1E). In addition, cell cycle analysis indicated that brown adipocytes rapidly exit the cell cycle from E16.0 to E17.5 (Figure 1F,G). Next, we consolidated gene expression across the nuclei from each developmental timepoint to form a pseudobulk snRNA-seq dataset and integrated it with our own bulk RNA-seq data from E15.5-P0.5 BAT. Analysis of the integrated dataset revealed a coordinated upregulation of genes canonically associated with brown adipocyte identity and function, including genes essential for thermogenesis, β-adrenergic signaling, and fatty acid metabolism (Figure 1H). By contrast, genes associated with proliferation and cell cycle functions decreased in expression over the same time span (Figure 1H). For an unbiased analysis of the gene expression changes over this developmental period, we performed Gene Set Enrichment Analysis (GSEA) by calculating single-sample enrichment scores for each of the 50 “hallmark” gene sets curated in the Molecular Signatures Database (MSigDB) [[46], [47], [48], [49], [50]]. The top upregulated pathways from E15 to E18 included adipogenesis, fatty acid metabolism, and oxidative phosphorylation, while the top downregulated pathways mainly related to cell division and proliferation (Figure 1I). Overall, these data demonstrate that the unique gene expression program of brown adipocytes is activated prenatally.

We next sought to identify adipose cell-extrinsic factors that drive the induction of the thermogenic gene program in late embryogenesis. In postnatal life, the cold-responsive induction of thermogenic genes and differentiation of new brown adipocytes is under the control of the SNS [3]. To characterize the sympathetic innervation of BAT during embryogenesis, we performed whole-mount immunostaining of tyrosine hydroxylase (TH) in chemically cleared BAT from E15.5 to E18.5 [51,52]. Co-staining of CD31, marking endothelial cells, served to visualize blood vessels. Based on the TH staining, the density of sympathetic nerve fibers increased dramatically from E15.5 to E18.5 (Figure 1J).

### 2.2 Genetic ablation of sympathetic nerves abrogates the postnatal BAT cold response

**Figure 2:**
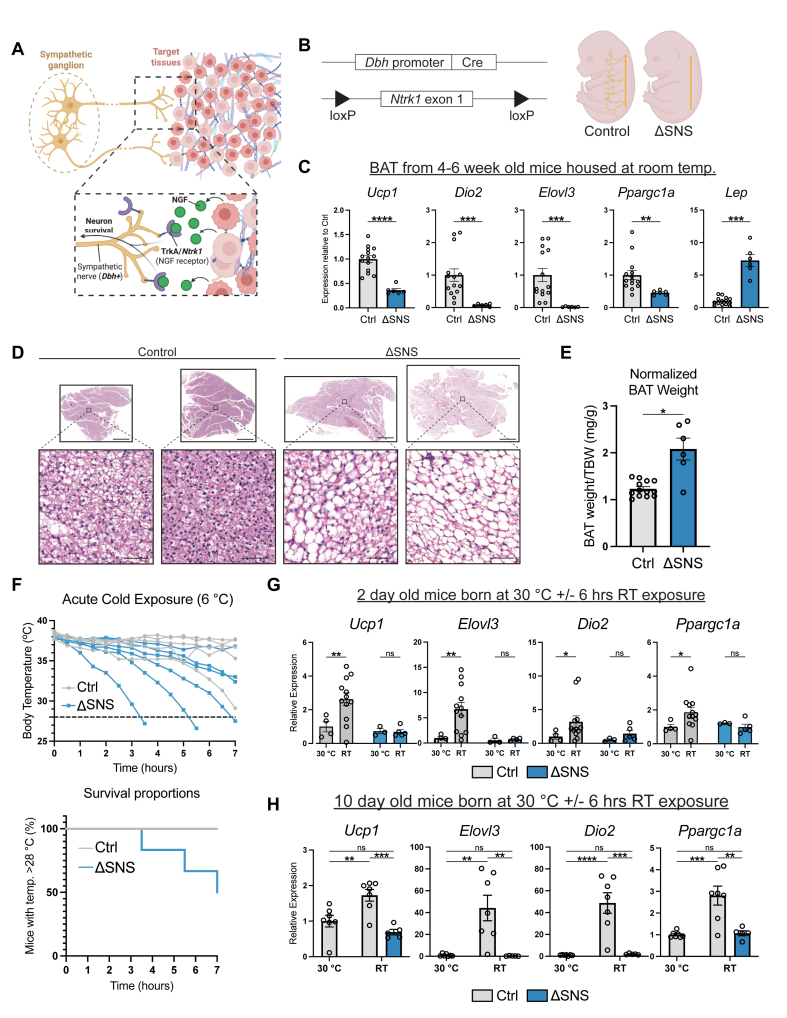
**Deletion of TrkA in sympathetic nerves disrupts BAT function (A)** Schematic illustrating NGF-TrkA signaling. Target tissue-derived NGF promotes axon branching and neuron survival via neuronal TrkA. **(B)** Schematic illustrating the design of the ΔSNS (*Dbh*^*Cre*^*Ntrk1*^*flox/flox*^) model. (Left) *Dbh*^*Cre*^ is used to delete exon 1 of *Ntrk1* (encoding TrkA) from sympathetic nerves. (Right) Development of sympathetic innervation is globally diminished in ΔSNS mice, from embryogenesis onwards. **(C**–**E)** mRNA expression levels (C), H&E staining (D), and total body weight (TBW)-normalized tissue weight (E) of BAT from 4 to 6 week old mice reared at room temperature (*n* = 6–14 per group; mean ± SEM). Scale bars: 1000 μm (tissue), 50 μm (inset). **(F)** Core body (colonic) temperature (top) and survival (bottom) in response to an acute cold challenge (6 °C) of 4-week-old mice reared at room temperature. Mice whose temperature dropped >10 °C relative to initial temperature were immediately euthanized. **(G**–**H)** mRNA expression levels of BAT from P2 (G) or P10 (H) mice kept at 30 °C since birth then either kept at 30 °C or exposed to room temperature (RT) for 6 h (*n* = 3–12 [P2] or 5–7 [P10] per group; mean ± SEM).

To examine the role of the SNS in BAT development, we generated mice lacking sympathetic nerves. To do this, we conditionally deleted *Ntrk1*, encoding TrkA, the high-affinity receptor for nerve growth factor (NGF) in dopamine β-hydroxylase (*Dbh*)-expressing neurons (Figure 2A,B) [53,54]. DBH is the last enzyme in the norepinephrine synthesis pathway and is more specific to sympathetic neurons than TH, which has been observed in (sensory) dorsal root ganglia in mice, including in neurons innervating some adipose tissues [[55], [56], [57], [58], [59]]. Without *Ntrk1*/TrkA, sympathetic nerves lack responsiveness to NGF and do not effectively innervate target tissues [40,[60], [61], [62]]. We refer to the *Dbh*^*Cre*^*Ntrk1*^*flox/flox*^ model as ΔSNS.

To assess the effectiveness of sympathetic nerve ablation in ΔSNS mice, we first analyzed BAT from adolescent control and ΔSNS mice reared at room temperature. We found that the expression of thermogenesis-related genes (*Ucp1*, *Elovl3*, *Dio2*, *Ppargc1a*) was lower in BAT of ΔSNS mice compared to controls, while the expression of leptin (*Lep*), a marker of white adipocyte identity, was higher (Figure 2C). Brown adipocytes also accumulated larger lipid droplets in ΔSNS mice compared to controls, likely reflecting decreased lipolytic activity (Figure 2D). The increased brown adipocyte size in ΔSNS mice was accompanied by an increase in tissue weight (Figure 2E). Functionally, ΔSNS mice failed to maintain their core body temperature in response to 6 °C cold exposure (Figure 2F). These results demonstrate that the degree of sympathetic nerve ablation in ΔSNS mice was sufficient to negatively impact the recruitment and physiologic function of BAT during cold exposure.

We also tested the ability of BAT from neonatal mice to respond to environmental cold by exposing 2- or 10-day-old pups to room temperature (∼22–24 °C) after being born at 30 °C. Room temperature represents a strong cold challenge for young mice. 2-day-old mice are not able to defend their body temperature under these conditions, while 10-day-old mice partially maintain it [63]. Exposure to room temperature for 6 h upregulated thermogenic genes (*Ucp1*, *Elovl3*, *Dio2*, *Ppargc1a*) in BAT from 2-day-old control pups compared to pups kept at 30 °C. This cold-induced gene response was abolished in the ΔSNS pups (Figure 2G). Similarly, room temperature exposure of 10-day-old control pups upregulated thermogenic gene expression in BAT, while BAT of cold-exposed ΔSNS pups resembled that of control pups kept at 30 °C (Figure 2H). These results indicate that the SNS is required for the cold-responsiveness of BAT in mice as young as 2 days old.

### 2.3 Prenatal differentiation of BAT is independent of sympathetic innervation

**Figure 3:**
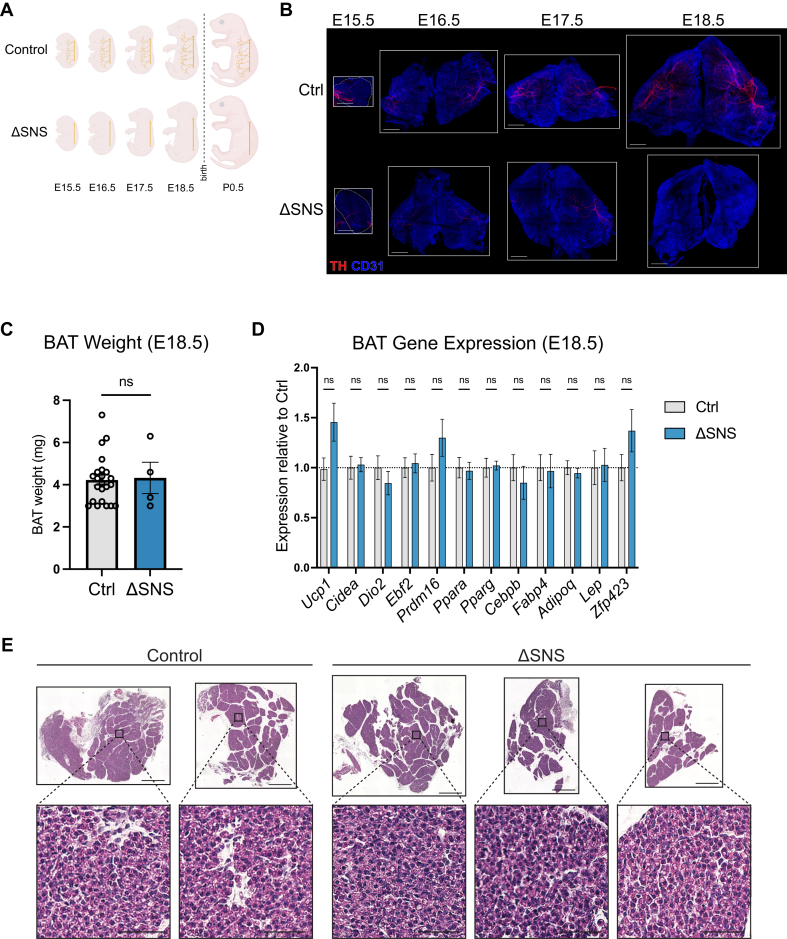
**Prenatal BAT development is independent of sympathetic innervation (A)** BAT was collected from E15.5-E18.5 embryos for whole-mount immunostaining. **(B)** Whole-mount immunostaining of TH (red) and CD31 (blue) in cleared BAT from E15.5-E18.5 embryos. Scale bars: 500 μm. **(C**–**E)** TBW-normalized tissue weight (C), mRNA expression levels (D), and H&E staining (E) of BAT from control or ΔSNS embryos at E18.5 (*n* = 8–16 per group; mean ± SEM). Scale bars: 500 μm (tissue), 50 μm (inset).

We next investigated whether BAT development during embryogenesis requires sympathetic innervation. We dissected BAT from ΔSNS and littermate control embryos at E15.5-E18.5 (Figure 3A). Whole-mount TH staining of cleared BAT revealed almost complete ablation of sympathetic nerves in ΔSNS BAT at all timepoints (Figure 3B). The residual TH^+^ nerve fibers may represent TH^+^ sensory nerves, which have been observed in white adipose tissue [59]. Regardless, the remaining TH^+^ nerve fibers were extremely sparse and did not develop the dense branching structure necessary to innervate individual brown adipocytes (Figure 3B). At E18.5, BAT weights were comparable between control and ΔSNS embryos (Figure 3C). Notably, expression of key thermogenic genes (*Ucp1*, *Cidea*, *Dio2*) remained intact in BAT from E18.5 ΔSNS embryos (Figure 3D). BAT expression levels of brown adipocyte-defining transcription factors (*Ebf2*, *Prdm16*), markers of adipocyte identity (*Pparg*, *Cebpb*, *Fabp4*, and *Adipoq*), and white adipocyte genes (*Lep*, *Zfp423*) were not affected by SNS-deficiency (Figure 3D). BAT morphology, as assessed by H&E staining, appeared identical in ΔSNS embryos and littermate controls at E18.5 (Figure 3E). Overall, these results indicate that the thermogenic profile of BAT develops normally in the absence of sympathetic innervation.

### 2.4 G_s_-coupled signaling is dispensable for embryonic brown adipocyte development

**Figure 4:**
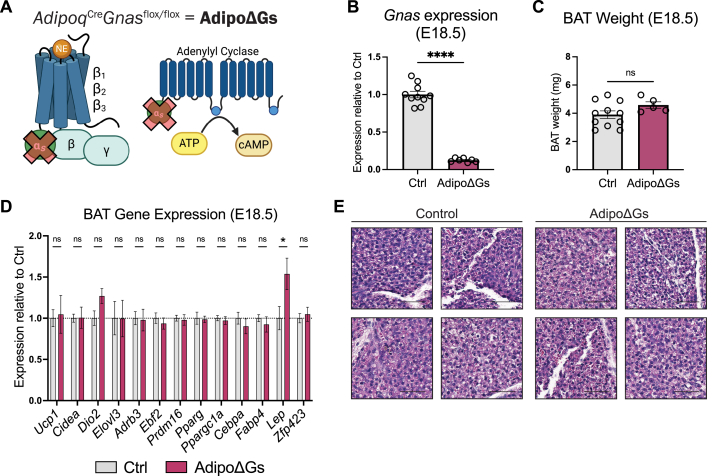
**Prenatal BAT development is independent of β-adrenergic and G_S_-coupled signaling (A)** Schematic illustrating adipocyte-specific G_s_ deletion in AdipoΔGs mice. **(B**–**E)** RT-qPCR confirmation of BAT *Gnas* knockout (B), TBW-normalized tissue weight (C), mRNA expression levels (D), and H&E staining (E) of BAT from control or AdipoΔGs embryos at E18.5 (*n* = 5–11 per group, mean ± SEM). Scale bars: 50 μm.

NE from maternal circulation partially rescues survival in embryos lacking the capacity to synthesize NE, indicating that some NE crosses the placenta [37]. In addition, epinephrine, which also stimulates adrenergic receptors, increases in concentration during late embryogenesis [37]. These observations raised the possibility that circulating catecholamines could support BAT differentiation, especially in the absence of sympathetic nerves. To test this idea, we conditionally deleted the α subunit of G_s_ (G_s_α), the G protein coupled to all three β-adrenergic receptors in adipocytes using *Adipoq*^*Cre*^*Gnas*^*flox/flox*^ (AdipoΔGs) mice [64,65]. Loss of G_s_α abolishes the signaling function of β-adrenergic receptors (Figure 4A).

*Gnas* mRNA levels were reduced by ∼90% in BAT of AdipoΔGs embryos at E18.5 relative to controls (Figure 4B). The small amount of residual *Gnas* expression (∼12% relative to littermate controls) likely derived from non-adipocyte cell types. We observed no difference in BAT weight relative to body weight in AdipoΔGs versus control embryos (Figure 4C). BAT from AdipoΔGs and control mice also showed equivalent expression levels of thermogenic genes (*Ucp1*, *Cidea*, *Dio2*, *Elovl3, Ppargc1a*), brown adipocyte fate-determining transcription factors (*Ebf2*, *Prdm16*), adipocyte markers (*Pparg*, *Cebpa*, *Fabp4*), and white adipocyte markers (*Lep*, *Zfp423*) (Figure 4D). Expression of the β_3_-adrenergic receptor (encoded by *Adrb3*) in BAT was unaffected by loss of *Gnas* (Figure 4D). H&E staining showed that BAT from AdipoΔGs embryos had normal cell and tissue morphology (Figure 4E). Together, these findings indicate that embryonic brown adipocytes develop independently of G_s_-coupled signaling from GPCRs, including β-adrenergic receptors.

### 2.5 Postnatal BAT develops independently of the SNS in the absence of cold stress

**Figure 5:**
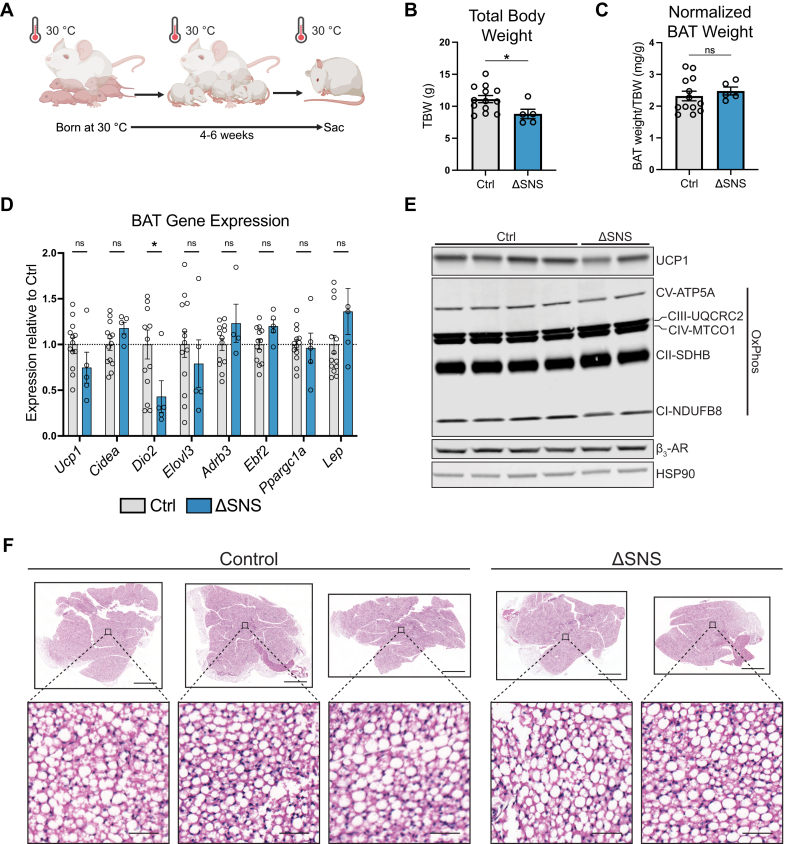
**Postnatal BAT development at 30 °C is independent of sympathetic innervation (A)** Pups were born and reared at 30 °C before sacrifice at 4–6 weeks. **(B**–**D)** Total body weight (TBW) (B), TBW-normalized BAT weight (C), and BAT mRNA expression levels (D) of 4–6 week old ΔSNS and control mice reared at 30 °C (*n* = 5–13 per group; mean ± SEM). **(E)** Immunoblot for UCP1, HSP90, and mitochondrial complexes in BAT from 4 to 6 week old ΔSNS and control mice reared at 33 °C. **(F)** H&E staining of BAT from 4 to 6 week old ΔSNS and control mice reared at 30 °C. Scale bars: 1000 μm (tissue), 50 μm (inset).

BAT continues to develop postnatally, growing in size due to continued differentiation of progenitor cells and increasing cell size from lipid accumulation [12,66,67]. In mice housed at room temperature, thermogenic gene expression increases during the first month of life [12]. However, the extent to which these changes are mediated by cold stress versus developmental processes was unclear. To reduce cold stress, we bred ΔSNS and control mice at 30 °C (Figure 5A). Although the true thermoneutral temperature for neonatal mice is higher than 30 °C [68], the ability to huddle with their dam and littermates likely further protected the pups from cold stress. We extracted BAT from ΔSNS mice and littermate controls at 4–6 weeks of age. ΔSNS mice exhibited a reduction in total body weight but no change in BAT weight normalized to body weight (Figure 5B,C). BAT from ΔSNS mice exhibited no difference in the expression levels of most thermogenesis-related genes (*Ucp1*, *Cidea*, *Elovl3*, *Adrb3*), nor *Ebf2* or *Lep* (Figure 5D). At the protein level, BAT from ΔSNS and control mice had comparable levels of UCP1, β_3_-adrenergic receptor (β_3_-AR), and electron transport chain complexes (Figure 5E). H&E staining showed that BAT from both ΔSNS and control mice accumulated large lipid droplets, which are characteristic of mice housed at a thermoneutral temperature (Figure 5F).

To further test whether β-adrenergic signaling or any other G_s_-coupled signaling pathways contribute to cold-independent BAT development postnatally, we bred AdipoΔGs and control mice at 30 °C (Fig. S1A). *Gnas* was knocked down in BAT with high efficiency (Fig. S1B). Total body weight and BAT weight were similar between the two groups at 4–6 weeks of age (Fig. S1C and D). The expression of thermogenic genes (*Ucp1*, *Cidea*, *Dio2*, *Elovl3*, *Ppargc1a*) and *Lep* showed no differences in BAT from AdipoΔGs versus control mice (Fig. S1E). Histologically, BAT from both AdipoΔGs and control mice had large lipid droplets, but AdipoΔGs BAT had more uniformly large lipid droplets and fewer cells with multiloculated lipid droplets, suggesting more lipogenic and/or less lipolytic activity compared to control BAT (Fig. S1F). In agreement with prior studies, BAT from AdipoΔGs mice reared at room temperature had larger lipid droplets, increased tissue weight, and decreased expression of thermogenic genes relative to controls (Fig. S1F-I) [69,70]. Overall, these results indicate that sympathetic innervation and G_s_-coupled signaling are necessary for the cold-responsiveness of BAT but not for its development under warm conditions.

### 2.6 Functional thermogenic capacity of BAT in genetically sympathectomized mice

**Figure 6:**
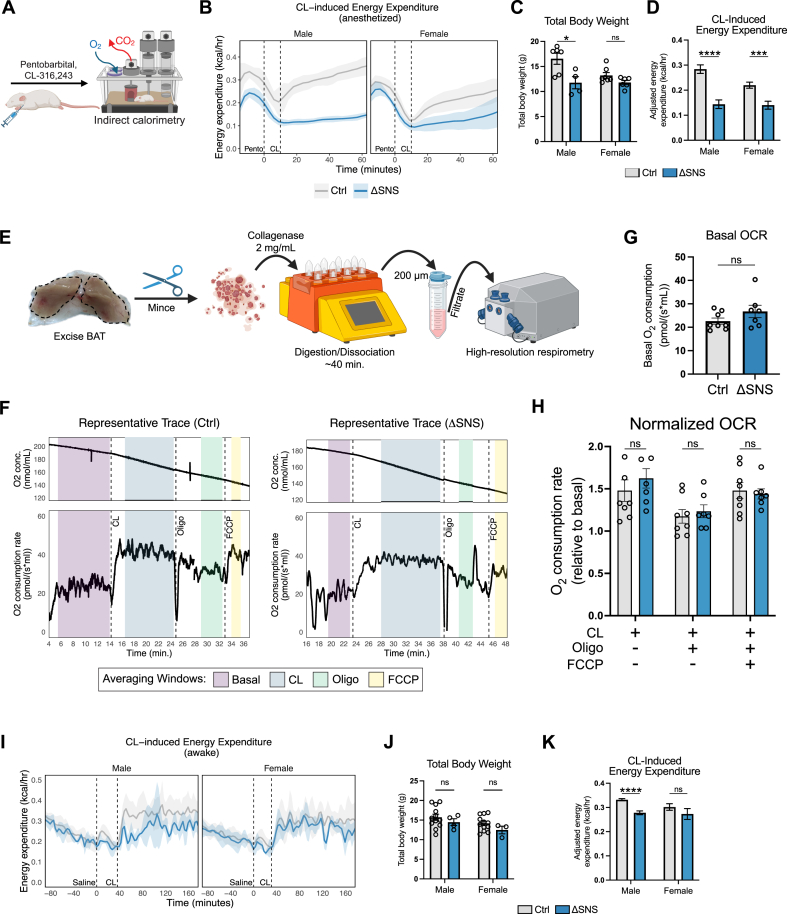
**Functional thermogenic capacity of BAT in genetically sympathectomized mice (A)** Indirect calorimetry was performed on mice anesthetized with pentobarbital then stimulated with CL-316,243 (CL). **(B**–**D)** Energy expenditure (EE) over time (B), total body weight (TBW) (C), and TBW-adjusted average CL-induced EE (D) of control and ΔSNS mice (*n* = 4–7 per group; mean ± SD for line graphs, mean ± SEM for bar graphs). Average TBW-adjusted CL-induced EE was calculated over the 30–50 min window. **(E)** Schematic illustrating the preparation of BAT cell suspensions. **(F)** Representative traces of oxygen concentration and oxygen consumption rate (OCR) of BAT cell suspensions sequentially treated with CL, oligomycin, and FCCP. **(G**–**H)** Basal OCR (G) and basal-normalized OCR (H) of BAT cell suspensions treated with indicated compounds (*n* = 7–8 per group; mean ± SEM). Values are averages from two runs per sample. **(I–K)** EE over time (I), TBW (J), and TBW-adjusted average CL-induced EE (K) of awake control and ΔSNS mice acclimated to metabolic cages, injected with saline, then stimulated with CL (*n* = 3–12 per group; mean ± SD for line graphs, mean ± SEM for bar graphs). Average TBW-adjusted CL-induced EE was calculated over the 40–180 min window.

We next sought to determine whether sympathetic innervation is required for BAT to develop its functional thermogenic capacity. The gold-standard method to measure BAT thermogenic function is to perform indirect calorimetry in mice anesthetized with pentobarbital and stimulated with either norepinephrine or the β_3_-selective adrenergic agonist CL-316,243 (CL) (Figure 6A) [71,72]. Anesthesia is used in these experiments to reduce the contribution of skeletal muscle activity (e.g., ambulation) to energy expenditure and to reduce inter-animal variability [71,73]. Interestingly, the elevation of energy expenditure in response to CL was markedly blunted in ΔSNS mice compared to controls (Figure 6B–D), despite these mice having equivalent expression of thermogenic genes and mitochondrial proteins in BAT (Figure 5D,E). The difference in CL-induced energy expenditure, adjusted for body weight, between ΔSNS and control mice was highly significant (Figure 6D). However, because the ΔSNS model diminishes sympathetic innervation globally, it was not immediately clear whether the reduction in CL-induced energy expenditure was due to a BAT-intrinsic or BAT-extrinsic defect.

To determine the intrinsic thermogenic capacity of BAT from ΔSNS and control mice reared at 30 °C, we performed high-resolution respirometry of BAT cell suspensions (Figure 6E). We obtained baseline readings followed by the sequential addition of CL, the ATP synthase inhibitor oligomycin, and the uncoupling agent carbonyl cyanide-p-trifluoromethoxyphenylhydrazone (FCCP) (Figure 6F). Each compound had its expected effect on the oxygen consumption rate (OCR): CL increased OCR above basal respiration; oligomycin reduced OCR, reflecting the loss of ATP-coupled respiration; and FCCP increased OCR, reflecting the uncoupling of the mitochondrial proton gradient from ATP production. We observed no significant OCR differences between the ΔSNS and control groups in any of the stages (Figure 6G,H). These results show that BAT from ΔSNS mice reared at 30 °C retains a normal capacity for CL-induced thermogenesis, and implies that the deficit in energy expenditure observed *in vivo* is caused by BAT-extrinsic physiological differences between ΔSNS and control mice.

The physiologic response to CL involves several organ systems. Although CL specifically targets the β_3_-adrenergic receptor on adipocytes, heart rate and respiratory rate increase upon CL administration [74]. The likely physiologic explanation is that CL-stimulated BAT consumes large amounts of oxygen, necessitating increased gas exchange and blood flow. Pentobarbital, like other anesthetics, is known to cause respiratory and circulatory depression [75,76]. We hypothesized that pentobarbital disproportionately affects ΔSNS mice, due to either decreased cardiopulmonary output at baseline, blunted upregulation of cardiopulmonary output in response to metabolic demand, or both. To test this hypothesis, we measured CL-induced energy expenditure in unanesthetized (awake) ΔSNS and control mice. To minimize confounding effects from behavioral factors, normally circumvented by the use of pentobarbital, mice were acclimated for ∼90 min and injected with saline before CL. Under these conditions, the CL-induced energy expenditure deficit of ΔSNS mice was largely attenuated, although the difference in male mice was still statistically significant (Figure 6I–K). These results suggest that the blunted response to CL observed in anesthetized ΔSNS mice (Figure 6B–D) is due to a confounding effect of the anesthesia rather than an intrinsic BAT defect. Together with the *ex vivo* respirometry (Figure 6F–H), these *in vivo* data support the conclusion that the functional thermogenic capacity of BAT develops independently of sympathetic innervation.

## 3 Discussion

BAT increases energy expenditure by dissipating energy as heat in response to cold-induced sympathetic activation. In this study, we found that the thermogenic identity and function of brown adipocytes develop independently of sympathetic signaling. BAT from genetically sympathectomized (ΔSNS) or adipocyte G_s_-deficient (AdipoΔGs) embryos had normal thermogenic gene expression, tissue size, and morphology relative to controls. Adolescent ΔSNS and AdipoΔGs mice exhibited normal BAT development when cold stress was eliminated. Sympathetic innervation was required for the physiologic response to CL in anesthetized mice but not for establishing the intrinsic thermogenic capacity of BAT. These findings are likely generalizable to other altricial species, such as rats, but not necessarily to immature or precocial species such as golden hamsters or guinea pigs.

It was initially surprising that anesthetized ΔSNS mice exhibited blunted energy expenditure in response to β-adrenergic stimulation. This method is considered the gold-standard assay for BAT thermogenic function [71,72]. However, the physiologic response to β-adrenergic stimulation is complex and involves multiple organ systems. Despite the absence of β_3_-adrenergic receptor expression in the heart and lungs, CL significantly increases heart rate and respiratory rate, likely due to increased oxygen demand in BAT [74,77]. Anesthetic agents, including pentobarbital, cause respiratory and circulatory depression in mice, just as they do in humans [75,76]. The neural pathways that mediate the response to hypoxia and hypercapnia involve post-ganglionic sympathetic neurons innervating the heart and lungs that are likely diminished in ΔSNS mice [60,77]. We speculate that the interaction of anesthesia and genetic sympathectomy results in an insufficient cardiopulmonary response to CL that could explain why anesthesia uncovers or accentuates a defect in CL-induced energy expenditure in ΔSNS mice.

In addition to its cardiopulmonary effects, CL also increases plasma insulin through an indirect effect on pancreatic β-cells [[78], [79], [80]]. Genetically sympathectomized mice (*Th*^*Cre*^*Ntrk1*^*flox/flox*^) similar to ΔSNS mice have disorganized pancreatic islets and impaired glucose-stimulated insulin secretion [40]. Although to our knowledge the functional role of insulin in CL-mediated thermogenesis has not been directly tested, it is possible that ΔSNS mice have a defect in this pathway that could be unmasked by anesthesia.

An important feature of this study was the use of two complementary genetic models. ΔSNS mice lack sympathetic innervation in BAT and likely many other organs [60]. Adipocytes in ΔSNS mice retain intact downstream signaling, allowing for the assessment of thermogenic function using β-adrenergic agonists. Similarly, circulating NE or epinephrine could compensate for the loss of direct sympathetic input to BAT in ΔSNS mice. In contrast, the AdipoΔGs model is cell type-specific and blocks downstream β-adrenergic signaling within adipocytes, eliminating the possibility of compensation by circulating catecholamines. However, the lack of β-adrenergic signaling in AdipoΔGs adipocytes precludes the analysis of NE–or CL-stimulated thermogenic function. While the AdipoΔGs model only blocks β-adrenergic signaling, the ΔSNS model also disrupts α-adrenergic signaling. In brown adipocytes, the contribution of α-adrenergic signaling alone has been found to be minimal, although it can potentiate the effect of β-adrenergic signaling [3,[81], [82], [83]].

In addition to NE, sympathetic nerves in BAT can also co-release neuropeptide Y (NPY) and ATP [84,85]. Recently, NPY has been implicated in the maintenance of healthy BAT and cold tolerance [86,87], and ATP signaling has been implicated in stress-induced BAT activation [88]. These co-transmitters are lost due to the ablation of sympathetic nerves in ΔSNS mice. Thus, an additional conclusion of this study is that neither NPY nor ATP are necessary for BAT development.

Deletion of G_s_ blocks many signaling pathways in addition to the β-adrenergic pathway. The list of G protein-coupled receptors (GPCRs) primarily coupled to G_s_ includes several that have demonstrated roles in BAT, such as the adenosine receptors A2A and A2B [89,90], the bile acid receptor TGR5/GPBAR [91], and the constitutively active lipolysis-responsive GPR3 [92]. Other G_s_-coupled GPCRs include receptors for glucagon, glucagon-like peptide 1 (GLP-1), gastric inhibitory peptide (GIP), parathyroid hormone (PTH), and thyroid stimulating hormone (TSH) [93]. The ability of BAT from AdipoΔGs mice to develop normally indicates that no G_s_-coupled GPCRs are essential for BAT development in the absence of cold stress.

In summary, the findings presented herein demonstrate that the thermogenic identity and functional thermogenic ability of BAT are established and maintained through SNS-independent mechanisms in late embryogenesis and early postnatal development. These results highlight the potential of harnessing cell-autonomous pathways to stimulate BAT development or function. Future studies aimed at identifying the molecular drivers of this intrinsic thermogenic programming may yield new targets for the therapeutic activation of BAT to ameliorate metabolic disease.

## 4 Methods

### 4.1 Mice

All animal experiments were performed according to procedures approved by the University of Pennsylvania’s Institutional Animal Care and Use Committee (approval no. 805649). Mice were housed under the care of University of Pennsylvania University Laboratory Animal Resources. Mice were raised either at room temperature or in a 30 °C incubator with a 12-h light–dark cycle and fed a regular chow diet (LabDiet, 5010 or 5053). Timed-pregnant Swiss Webster mice (CFW, Charles River) were used for the E15.5-P25 time-course RNA-seq, RT-qPCR, and H&E staining. *Dbh*^*Cre*^ [54] and *Ntrk1*^*flox*^ [53] mice were obtained from Dr. Rejji Kuruvilla (Johns Hopkins University) on a C57BL/6 background and bred to generate *Dbh*^*Cre*^*Ntrk1*^*flox/flox*^ (ΔSNS) mice and littermate controls (*Dbh*^*Cre*^*Ntrk1*^*flox*/+^, *Ntrk1*^*flox/flox*^, *Ntrk1*^*flox*/+^). No differences were observed between control genotypes in any experiment. *Adipoq*^*Cre*^ [64] mice on a C57BL/6J background were obtained from The Jackson Laboratory (strain #028020). *Gnas*^*flox*^ [65] mice were obtained from Dr. Eileen Shore (University of Pennsylvania) on a C57BL/6 background and were bred with *Adipoq*^*Cre*^ mice to generate *Adipoq*^*Cre*^*Gnas*^*flox/flox*^ (AdipoΔGs) mice and littermate controls (*Gnas*^*flox/flox*^). For embryonic studies, timed matings were set up at night and plugs were checked in the morning (E0.5). Embryos were collected for analysis mid-day on the specified day. For experiments in which mice were born at 30 °C, pregnant dams were transferred to incubators prior to delivery. Experiments were conducted on both male and female mice unless otherwise specified.

### 4.2 Acute exposure to cold or room temperature

For acute room-temperature exposure, 2- or 10-day-old mouse pups were placed individually in air-exposed plastic dishes without lids for the specified duration prior to tissue collection. For acute cold exposure, 4-week-old mice were transferred to individual cages in an incubator maintained at 6 °C. Mice were fasted for the 7-hour duration of the experiment. Core body temperature was measured every 30 min with a colonic probe. Mice whose body temperatures dropped >10 °C relative to initial temperature were immediately euthanized.

### 4.3 Indirect calorimetry

Indirect calorimetry was performed using a Promethion CORE system (Sable Systems) maintained by the University of Pennsylvania Rodent Metabolic Phenotyping Core (RRID:SCR_022427). Mice were placed in individual metabolic cages in a temperature-controlled cabinet (Sable Systems) set to 33 °C. In experiments involving anesthesia, mice were injected intraperitoneally with 75 mg/kg pentobarbital, followed by interscapular subcutaneous injection with 1 mg/kg CL-316,243 (Sigma #C5976) at the specified timepoints. In experiments without anesthesia, mice were allowed to acclimate to the metabolic cages for 90 min prior to injection with saline, followed by injection with 1 mg/kg CL-316,243. Metabolic parameters were calculated automatically using manufacturer-provided software (Promethion Live v23.0.10). Data were aligned to set the time of first injection to 0 and interpolated to estimate metabolic parameters at 3-minute intervals.

### 4.4 Preparation of BAT cell suspension

Mice were euthanized by cervical dislocation. BAT was immediately dissected and kept in warm DMEM/F12 (Gibco #11330032) prior to digestion. The two BAT pads from each mouse were placed in a gentleMACS C tube (Miltenyi Biotech pre-coated with 1 mL DMEM/F12 + 2% bovine serum albumin (BSA). Tissues were minced with scissors until no pieces larger than 2–3 mm in diameter remained. Tissues were digested/dissociated in 3 mL digestion medium (DMEM/F12, 2% BSA, 2 mg/mL Collagenase Type 1 (Worthington Biochemical), 1 mg/mL soybean trypsin inhibitor (Gibco #17075029), 10 mM glucose) using a gentleMACS Octo Dissociator (Miltenyi Biotec) on program “37C_mr_ATDK-1”. The resulting mixture was then poured over a 200 μm filter into a 25 mL conical tube to remove debris. BAT cell suspensions were used immediately for high-resolution respirometry.

### 4.5 High-resolution respirometry

High-resolution respirometry was carried out using an Oroboros O2k (Oroboros Instruments). 1 mL of BAT cell suspension was added to 1 mL DMEM/F12 in each chamber. Chamber stir bars spun continuously at 500 rpm. For each run, cells were successively treated with CL-316,243 (1.25 μM), oligomycin (1 μM), and FCCP (10 μM). The oxygen consumption rate (OCR) was allowed to stabilize prior to the addition of each compound via Hamilton syringe. In optimization experiments, additional injections of each compound did not further affect OCR, and addition of 1 μM norepinephrine after CL-3162,43 did not further increase OCR. Each sample was analyzed in two separate runs. For each run, one control and one experimental sample were analyzed side-by-side, one in each of the instrument’s two chambers. The chambers assigned to the control and experimental samples were swapped for the second run. After all the data were collected, steady-state OCR values were calculated for each stage and normalized to the basal state. Technical replicates for each sample were averaged.

### 4.6 RT-qPCR

Tissues were lysed in TRIzol (Invitrogen #15596018) using a TissueLyser II (QIAGEN) for 4 min at 24 Hz in pre-chilled tube adapters. Total RNA was isolated according to the provided TRIzol protocol, and RNA concentration was quantified using a NanoDrop spectrophotometer. mRNA was reverse-transcribed to cDNA using the High-Capacity cDNA Reverse Transcription Kit (Applied Biosystems #4368814). Real-time PCR was performed on a QuantStudio 5 Real-Time PCR system (Applied Biosystems) using PowerUp SYBR Green Master Mix for qPCR (Applied Biosystems #A25742). Fold changes were calculated using the ΔΔCT method, with TATA-binding protein (*Tbp*) mRNA serving as a normalization control.

### 4.7 Oligonucleotides used in this study

.

**Table:** 

qPCR primer	Sequence (5′-3′)
*Adipoq* F	GCACTGGCAAGTTCTACTGCAA
*Adipoq* R	GTAGGTGAAGAGAACGGCCTTGT
*Adrb3* F	CAGCCAGCCCTGTTGAAG
*Adrb3* R	CCTTCATAGCCATCAAACCTG
*Cebpa* F	TGCGCAAGAGCCGAGATAA
*Cebpa* R	CGGTCATTGTCACTGGTCAACT
*Cebpb* F	ACGACTTCCTCTCCGACCTCT
*Cebpb* R	CGAGGCTCACGTAACCGTAGT
*Cidea* F	CCTTAAGGGACAACACGCATT
*Cidea* R	GCCTGTATAGGTCGAAGGTGA
*Dio2* F	CAGTGTGGTGCACGTCTCCAATC
*Dio2* R	TGAACCAAAGTTGACCACCAG
*Ebf2* F	GCTGCGGGAACCGGAACGAGA
*Ebf2* R	ACACGACCTGGAACCGCCTCA
*Elovl3* F	TCCGCGTTCTCATGTAGGTCT
*Elovl3* R	GGACCTGATGCAACCCTATGA
*Fabp4* F	ACACCGAGATTTCCTTCAAACTG
*Fabp4* R	CCATCTAGGGTTATGATGCTCTTCA
*Gnas* F	AACAGTAAGACCGAGGACCAG
*Gnas* R	CTTCACAATGGTGCTTTTGCC
*Lep* F	CAGACAGAGCTGAGCACGAAA
*Lep* R	CTGCACCCTATGTCACCATCA
*Ppara* F	GCGTACGGCAATGGCTTTAT
*Ppara* R	GAACGGCTTCCTCAGGTTCTT
*Pparg* F	AGGGAGTTCCTCAAAAGCCTG
*Pparg* R	AGGTTGTCTTGGATGTCCTCG
*Ppargc1a* F	CCCTGCCATTGTTAAGACC
*Ppargc1a* R	TGCTGCTGTTCCTGTTTTC
*Prdm16* F	CAGCACGGTGAAGCCATTC
*Prdm16* R	GCGTGCATCCGCTTGTG
*Ucp1* F	CAGAAGGATTGCCGAAACTGT
*Ucp1* R	ATCTTGTTTCCGAGAGAGGCA
*Zfp423* F	AGTGCCCCGTCTGCTTCACAG
*Zfp423* R	CTGCGGAGAGGTGTCCTGTCG

### 4.8 RNA sequencing

Tissues were lysed as for RT-qPCR. Total RNA of BAT from E15.5-E18.5 embryos and P0.5 neonates was isolated using TRIzol and purified using PureLink RNA Mini columns (Invitrogen #12183025). RNA sequencing was performed by Azenta (Indianapolis, IN, USA) as follows: RNA samples were quantified using Qubit 3.0 Fluorometer (Life Technologies, Carlsbad, CA, USA) and RNA integrity was checked using Agilent TapeStation 4200 (Agilent Technologies, Palo Alto, CA, USA). ERCC RNA Spike-In Mix 1 (Cat: #4456740) from ThermoFisher Scientific was added to normalized RNA samples before initiating library preparation following the manufacturer’s protocol. RNA sequencing libraries were prepared using the NEBNext Ultra II RNA Library Prep Kit for Illumina and the NEBNext Poly(A) mRNA Magnetic Isolation Module following the manufacturer’s instructions (NEB, Ipswich, MA, USA). Briefly, mRNAs were initially enriched with Oligod(T) beads. Enriched mRNAs were fragmented for 15 min at 94 °C. First-strand and second-strand cDNA were subsequently synthesized. cDNA fragments were end-repaired and adenylated at 3′ ends, and universal adapters were ligated to cDNA fragments, followed by index addition and library enrichment by PCR with limited cycles. The sequencing library was validated on the Agilent TapeStation (Agilent Technologies, Palo Alto, CA, USA), and quantified by using Qubit 2.0 Fluorometer (Invitrogen, Carlsbad, CA) as well as by quantitative PCR (KAPA Biosystems, Wilmington, MA, USA). The sequencing libraries were clustered on a flowcell. After clustering, the flowcell was loaded on the Illumina NovaSeq 6000 instrument according to the manufacturer’s instructions. The samples were sequenced using a 2 × 150 bp Paired-End (PE) configuration, targeting approximately 25 M reads/sample. The control software conducted image analysis and base calling. Raw sequence data (.bcl files) generated by the sequencer were converted into fastq files and de-multiplexed using Illumina’s bcl2fastq 2.20 software. One mismatch was allowed for index sequence identification.

### 4.9 Bioinformatics analysis

#### 4.9.1 snRNA-seq analysis

snRNA-seq count data from an atlas of murine embryonic development were downloaded [45]. Data from cells in the adipocyte lineage (annotated as “Adipocyte progenitor cells”, “Adipocyte cells (Cyp2e1+)”, and “Brown adipocyte cells”) from E15.0-P0 embryos were extracted for further analysis using Seurat v4 [94]. After log-normalization, cells were scored for S-phase and G2M-phase gene expression (S.Score, G2M.Score) using the CellCycleScoring function. Data for the 2500 most variable genes were scaled using ScaleData, using S.Score and G2M.Score as covariates. Dimensionality reduction was performed using principal component analysis with the top 30 principal components. Cell–cell similarity graphs were constructed using FindNeighbors, and clustering was performed with FindClusters at a resolution of 0.5. Nuclei with >0.3% reads mapping to mitochondrial genes or belonging to clusters defined by expression of white adipocyte marker genes (*Lep*, *Retn*) were excluded, and the above processing steps were repeated to produce the final refined dataset of 52,100 nuclei from E15.0-E18.75. Count data from each embryonic stage were aggregated using AggregateExpression to produce a pseudobulk snRNA-seq dataset.

#### 4.9.2 RNA-seq analysis

Reads were pseudoaligned to the mouse genome (GRCm39), and transcript abundances were quantified using kallisto v0.46.2 with default parameters [95]. RNA abundances were converted to counts using tximport with the “lengthScaledTPM” option [96].

#### 4.9.3 RNA-seq & pseudobulk snRNA-seq integrative analysis

RNA-seq and pseudobulk snRNA-seq count data were normalized using the trimmed mean of M values (TMM) method [97] as implemented in edgeR [98]. Gene set enrichment analysis (GSEA) and single-sample GSEA (ssGSEA) were performed on log-transformed expression values using the clusterProfiler and GSVA R packages [[46], [47], [48],^99^]. Gene sets were downloaded from the Molecular Signatures Database (MSigDB) [49,50,100]. Heatmaps were generated using the ComplexHeatmap R package [101].

### 4.10 Histology

Tissues were fixed in 4% paraformaldehyde overnight, washed in PBS, dehydrated in ethanol, paraffin-embedded and sectioned. Slides were stained with hematoxylin and eosin (H&E) and imaged on a KEYENCE BZ-X700 microscope using the 10X or 20X objective.

### 4.11 Whole-mount immunostaining and tissue clearing

Tissues were processed using the Adipo-Clear protocol [51,52] with minor modifications. Briefly, tissues were fixed in 4% paraformaldehyde overnight, washed in PBS, dehydrated in a gradient of methanol in B1n buffer (0.3 M glycine, 0.1% v/v Triton X-100, 0.1% w/v sodium azide, pH 7), delipidated in dichloromethane, bleached in 5% H_2_O_2_/methanol overnight, rehydrated in a gradient of methanol in B1n buffer, and washed in PTxwH buffer (1x PBS, 0.1% v/v Triton X-100, 0.05% v/v Tween 20, 2 μg/mL heparin, 0.01% w/v sodium azide). Tissues were then incubated for 3 days at room temperature with the following primary antibodies: rabbit anti-TH (1:200; Millipore #AB152) and goat anti-CD31 (2 μg/mL, R&D Systems #AF3628). After extensive washes with PTxwH buffer, tissues were incubated for 3 days at room temperature with the following secondary antibodies: Alexa Fluor 647-conjugated donkey anti-rabbit IgG (1:200; Invitrogen #A-31573) and Alexa Fluor 568-conjugated donkey anti-goat IgG (1:200; Invitrogen #A-11057). Tissues were again extensively washed with PTxwH buffer. Tissues were then embedded in 2% low melting temperature SeaPlaque agarose (Lonza #50101), dehydrated in a gradient of methanol in water, incubated in dichloromethane, and cleared in dibenzyl ether. Samples were mounted on slides in dibenzyl ether using 9 mm FastWells incubation chambers (Electron Microscopy Sciences #70325-50) and imaged on a Leica Mica confocal microscope.

### 4.12 Immunoblotting

BAT was homogenized in RIPA buffer (150 mM NaCl, 1% NP-40, 0.1% sodium deoxycholate, 0.1% SDS, 100 μm Tris–HCl, pH 7.4) supplemented with cOmplete Protease Inhibitor Cocktail tablets (Roche #11836170001), Halt Phosphatase Inhibitor (Thermo Scientific #78420), and 1 mM PMSF (Sigma #93482) using a TissueLyser II (QIAGEN) for 2 min at 22 Hz in pre-chilled tube adapters. Lysates were then centrifugated at 21,130×*g* for 15 min at 8 °C to allow free lipid to float. Resuspended precipitates and the non-lipid fraction of the supernatants were transferred to new tubes and sonicated using a Bioruptor Sonicator (Diagenode UCD-200) at high amplitude for 2 min (30 s on/30 s off). Lysates were centrifugated at 21,130×*g* for 10 min at 4 °C, and supernatants were transferred to a new tube. Protein concentrations were determined using the Pierce BCA Protein Assay Kit (Thermo Scientific #23225). Samples were diluted in 4X NuPAGE LDS Sample Buffer (Thermo Scientific #NP0007) with 10% β-mercaptoethanol (final concentration 2.5%) and adjusted with 1X sample buffer in RIPA buffer to equalize protein concentrations. Proteins were denatured at 95 °C for 5 min, except for samples used for OxPhos immunoblotting. Samples were separated on NuPAGE Novex 4–12% Bis-Tris gels (Invitrogen #NP0335BOX) and transferred to PVDF membranes (Invitrogen #IB24002) using an iBlot 2 Gel Transfer Device (Thermo Fisher Scientific #IB21001). Membranes were blocked in 50% Odyssey Blocking Buffer (LI-COR # 927–50000) in TBST (TBS, 0.1% Tween 20), incubated overnight with primary antibody at 4 °C, washed with TBST, incubated for 1 h in secondary antibody at room temperature, washed again with TBST, and imaged using a LI-COR Odyssey Imager. Primary antibodies used were anti-UCP1 (1:1000; R&D Systems #MAB6158), anti-HSP90 (1:1000; Cell Signaling Technology #4874), and total OXPHOS rodent antibody cocktail (1:1000; Abcam #ab110413).

### 4.13 Statistical analysis

For comparisons between two groups, Welch’s t-tests were performed. For comparisons between more than two groups, one-way ANOVAs with pairwise comparisons corrected with Holm’s method [102] were performed. For indirect calorimetry experiments, energy expenditure was analyzed by analysis of covariance (ANCOVA) with genotype and sex as factors and total body weight as a covariate. Post hoc pairwise comparisons between genotypes were performed separately within each sex. All statistical analyses were performed using the R programming language. For visualization, *p*-values are represented as follows: ∗*p* < 0.05, ∗∗*p* < 0.01, ∗∗∗*p* < 0.001, ∗∗∗∗*p* < 0.0001.

## CRediT authorship contribution statement

**Ethan C. Fein:** Writing – review & editing, Writing – original draft, Visualization, Methodology, Investigation, Funding acquisition, Formal analysis, Data curation, Conceptualization. **Sarmistha Mukherjee:** Methodology. **Joseph A. Baur:** Resources, Funding acquisition. **Patrick Seale:** Writing – review & editing, Writing – original draft, Supervision, Resources, Funding acquisition, Conceptualization.

## Funding

This work was supported by 10.13039/100000002NIH grants R01 DK121801 and R01 DK120982 to P.S.; and 10.13039/100000002NIH grant F30DK138742 to E.C.F.

## Declaration of competing interest

The authors declare the following financial interests/personal relationships which may be considered as potential competing interests: Patrick Seale reports financial support was provided by 10.13039/100000002National Institute of Diabetes and Digestive and Kidney Diseases. Ethan C. Fein reports financial support was provided by 10.13039/100000016National Institutes of Health. If there are other authors, they declare that they have no known competing financial interests or personal relationships that could have appeared to influence the work reported in this paper.

## Data Availability

Data will be made available on request.
