# Supplementary material for: The innate thermogenic capacity of brown adipose tissue develops independently of sympathetic signaling
Source: Mol Metab. 2025 Dec 9;103:102299. doi: 10.1016/j.molmet.2025.102299 (PMC12794445; doi:10.1016/j.molmet.2025.102299)
Supplement: Multimedia component 1 [file mmc1.pdf]

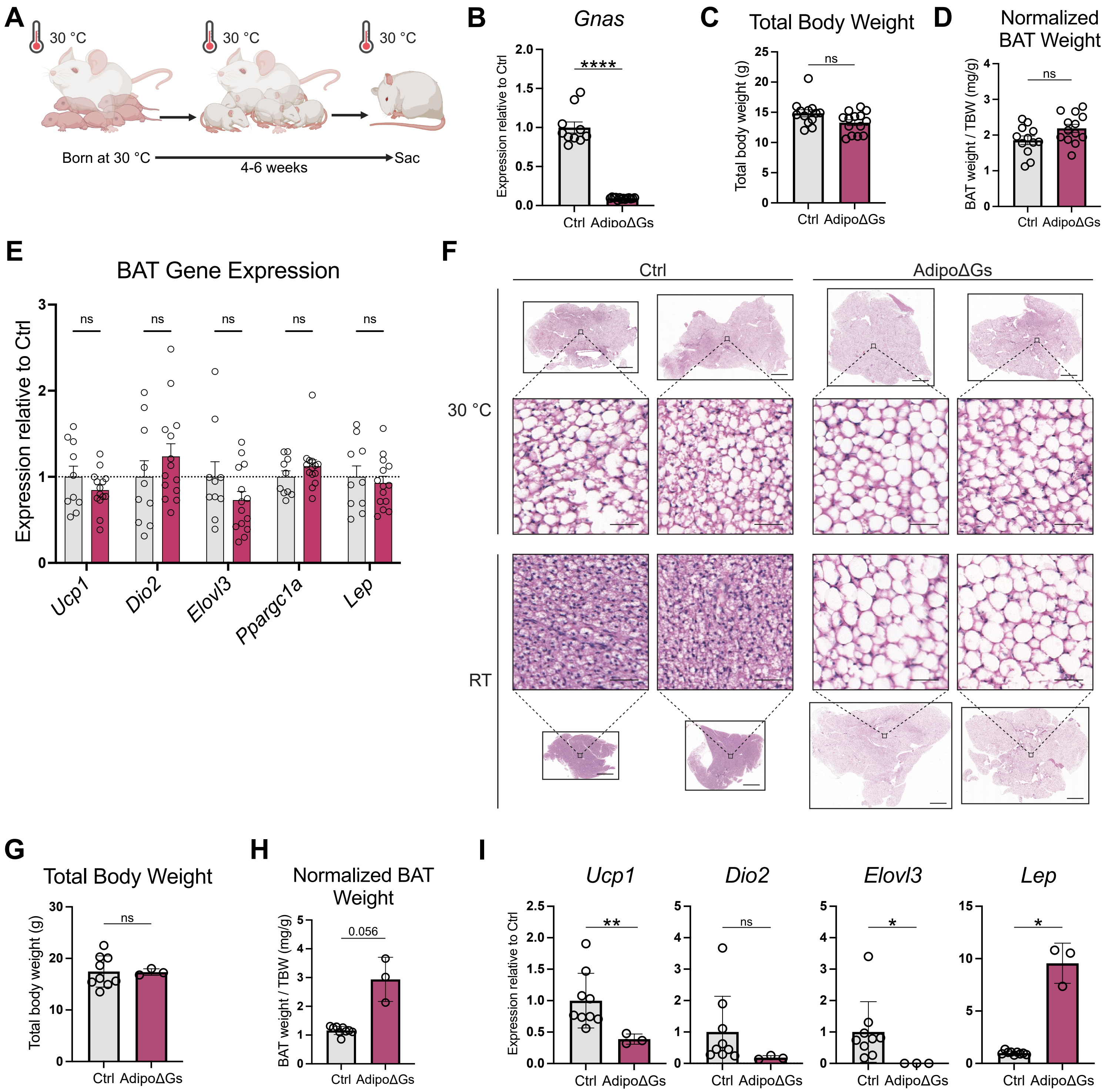

Figure S1: Postnatal BAT development at 30°C is independent of GS-coupled signaling.

(A) Pups were born and reared at 30°C before sacrifice at 4-6 weeks. **(B-E)** RT-qPCR confirmation of BAT *Gnas* knockout (B), total body weight (TBW) (C), TBW-normalized BAT weight (D), and BAT mRNA expression levels (E) of 4–6-week-old control and *AdipoΔGs* mice reared at 30°C (n = 10-14 per group; mean ± SEM). **(F)** H&E staining of BAT from 4–6-week-old control and *AdipoΔGs* mice reared at room temperature (RT) or 30°C. Scale bars: 1000 µm (tissue), 50 µm (inset). **(G-I)** TBW (G), TBW-normalized BAT weight (H), and BAT mRNA expression levels (I) of 4–5-week-old control and *AdipoΔGs* mice reared at RT (n = 3-9 per group; mean ± SEM).
